# Recent Insights into *Escherichia coli* and *Vibrio* spp. Pathogenicity and Responses to Stress

**DOI:** 10.3390/microorganisms10010038

**Published:** 2021-12-26

**Authors:** Vladimir R. Kaberdin, Inés Arana

**Affiliations:** 1 Department of Immunology, Microbiology and Parasitology, Faculty of Science and Technology, University of the Basque Country UPV/EHU, 48940 Leioa, Spain; 2 Research Centre for Experimental Marine Biology and Biotechnology (PIE-UPV/EHU), 48620 Plentzia, Spain; 3 IKERBASQUE, Basque Foundation for Science, Maria Diaz de Haro 3, 48013 Bilbao, Spain

The ubiquitous presence of microorganisms is largely attributed to their tremendous capacity to successfully adapt and survive in highly adverse environments. The main goal of the current collection of reports and its continuation in the second edition of the Special Issue “Bacterial Responses to Environmental Stress and Their Specific Contribution to *Escherichia coli* and *Vibrio* spp. Survival and Virulence” is to present recent findings illustrating the key adaptation mechanisms and strategy exploited by well-known gamma-proteobacteria, *Escherichia coli* and *Vibrio* spp., in order to thrive in different environments and elicit infections.

The first, *E. coli*, is arguably the best characterized gamma-proteobacterium [1], which remains a popular model organism to study the adaptation and host–pathogen interactions of enterobacteria. The latter play an essential role in human and animal microbiota. In particular, many enterobacteria (e.g., *E. coli*, *Klebsiella*, and *Shigella*) are notorious for causing severe infections and for their ability to easily acquire resistance to a large number of drugs currently used to treat diseases. Therefore, it does not come as a surprise that the continuous spread of multidrug-resistant strains and their persistence during infection and disease development pose a true challenge for the current healthcare system, thus making it important to study the genetic bases and clinical manifestations of multidrug-resistant bacteria. Some articles included in the first and second edition of this Special Issue highlight significant progress made in this field. In particular, it concerns further advances achieved in addressing the contribution of multidrug-resistant *E. coli* strains to the development of intestine infection [2] and some health disorders [3,4]. Other reports provide a number of interesting insights into the mechanisms of pathogenesis by addressing the role of colicins [5] and bacterial toxins [6] during the infection process and by providing insights into various adaptation strategies [7,8,9,10].

Unlike members of the Enterobacteriaceae family, *Vibrio* spp. have received particular attention as model organisms widely employed to study the impact of climate change on the dynamics, spread and pathogenicity of microbial species that inhabit aquatic systems. Although many *Vibrio* spp. are not pathogenic, some of them do cause severe diseases in humans and a large number of marine organisms [11]. Apparently, the best-known one is cholera, caused by pathogenic *V. cholerae* strains whose recent spread around the globe was largely attributed to global warming. Due to the characteristic responses of vibrios to climate change, which were documented in numerous studies, a number of *Vibrio* spp. have recently been referred to as microbial barometers, thus emphasizing their ability to sense and respond to global warming and associated environmental changes [12].

Owing to the important ecological roles of *Vibrio* spp., there is increasing interest in the isolation and characterization of new members of the *Vibrio* genus. One of the recent studies employing high-throughput sequencing techniques and gene annotation revealed a new species, *V. fujianensis*, that possesses a unique environmental adaptability [13]. This finding makes this new bacterium an attractive model organism to study adaptation mechanisms. In fact, some of them (e.g., cell adaptation to visible light) are still poorly understood, and a new study by Orruño et al. [14] certainly helps to gain new insights regarding the impact of this environmental factor on cell survival and adaptation.

In addition to physical factors, the nutrient content of marine ecosystems can likewise influence the physiology and lifestyles of *Vibrio* spp. The study of Liu et al. [15] nicely illustrates this fact by addressing the impact of the global regulator CsrA on *V. alginolyticus* motility and carbohydrate metabolism. The utilization of some natural biopolymers is assisted by hydrolytic enzymes secreted by *Vibrio* spp. The latter include diverse chitinases, a group of enzymes essential for recycling of different forms of chitin naturally present in the exoskeleton of many marine organisms. The study, which was carried out by Debnath et al. [16], provides new insights regarding the role of chitin-dependent growth and its impact on the natural competence of *V. parahaemoliticus*.

Besides the high adaptability of pathogens and the ongoing expansion of their habitats due to climate change, another important factor that determines their spread and the increase in incidence of infections is horizontal gene transfer (HGT), which is often mediated by plasmids. Two articles in this Special Issue are focused on *E. coli* and *V. parhaemoliticus* plasmids [17,18] that harbor gene coding for multidrug resistance and virulence, respectively.

In conclusion, we hope that the content of this Special Issue will serve as an important source of information and will stimulate further interest in using *E. coli* and *Vibrio* spp. to address the fundamental and timely questions concerning the spread of pathogenic species and their impact on human health.
